# Cytomegalovirus Infection following Kidney Transplantation: a Multicenter Study of 3065 Cases

**Published:** 2012-05-01

**Authors:** B. Einollahi

**Affiliations:** *Nephrology and Urology Research Center, Baqiyatallah University of Medical Sciences, Tehran, Iran *

**Keywords:** Cytomegalovirus infection, Kidney transplantation, Incidence, Risk factor

## Abstract

**Background:** Cytomegalovirus (CMV) infection is a common complication following kidney transplantation.

**Objective:** To assess the incidence and risk factors of CMV infection among renal transplant recipients.

**Methods:** In a retrospective multicenter study, 3065 renal transplant recipients from 17 transplant centers of Iran were studied between April 2008 and January 2011. Kidney transplant patients were routinely monitored by sequential blood samples drawn for use in the CMV-pp65 antigenemia assay, and for hematological and biochemistry tests.

**Results: **63% of studied patients were males; the mean±SD age of participants was 38±15 years. The majority of cases (81%) received a kidney from a living unrelated donor (LURD), 9% from living related donor (LRD), and 10% from deceased donors. 671 patients experienced CMV viremia. The incidence of CMV infection was 21.9% (95% CI: 20.4%–23.4%). The rate was higher in the first 6 months after transplantation (p<0.001); in recipients with higher level of cyclosporine (p<0.001); in those with lower hemoglobin concentration (p=0.02); patients with elevated ALT (p<0.001); those with increased fasting blood sugar (p=0.005); recipients with dyslipidemia (p<0.05); deceased kidney recipients (p=0.006); and patients with kidney graft impairment (p=0.01). In multivariate regression analysis, time since kidney transplantation (p<0.001) and renal allograft failure (p<0.001) were the only risk factors associated with CMV infection.

**Conclusions:** CMV infection was a common complication in the first 6 months of kidney transplantation, particularly among patients with kidney graft impairment.

## INTRODUCTION

Despite the recent advancements in immunosuppressive regimens that have led to increased survival of renal recipients, there are considerable risks of developing infectious complications. Cytomegalovirus (CMV) is the major cause of infectious disease after kidney transplantation [1]. It is also the leading cause of morbidity and mortality in organ transplant recipients [2].

Because of its opportunistic behavior under immunosuppression, active CMV infections generally have a large impact on the clinical course of organ transplant recipients. The detrimental effect of CMV infection on the outcomes of transplantation is beyond any doubt. In transplant patients, beside direct effects such as systemic and organ infection/disease, CMV has been associated to indirect effects, including enhanced systemic immunosuppression (*i.e*., effect favoring opportunistic infections) [1], acceleration of HCV infection, increased risk of post-transplant malignancies (*i.e.*, post-transplantation lymphoproliferative diseases) and the potential role in graft rejection [3,4]. For several years, a number of clinical studies have shown a probable association between CMV and acute organ rejection [5-7].

Diagnosis of CMV infection in renal transplant recipients should be done by isolation of the virus. In addition, detection of CMV-pp65 in peripheral blood leukocytes (antigenemia) has proved to be a simple, quick, sensitive, and economic test for the detection of CMV infection; it permits a same-day diagnosis. Moreover, the direct clinical manifestations of CMV infection usually occur one to four months after transplantation [4]. The objective of this study was therefore to examine the incidence of CMV infection and its risk factors in our renal transplant population.

## PATIENTS AND METHODS

This retrospective cohort study was carried out in a single laboratory center, referred blood samples for CMV-pp65 antigenemia assay (CMV Ag) from kidney transplant centers of Iran, to assess the prevalence and its risk factors among kidney transplant patients between April 2008 and January 2011.

Blood samples from 3065 renal transplant recipients (RTRs) who underwent in one of 17 transplant centers in Iran were analyzed. After renal transplantation, patients were routinely monitored by sequential blood samples drawn for CMV-pp65 antigenemia assay and hematological and biochemistry tests. All individuals and laboratory data were obtained by examination of the patients’ records at Gholhak diagnostic laboratory.

The patients’ blood samples for CMV-pp65 antigenemia assay were categorized into three groups according to the time since kidney transplantation—less than one month (6%), 2–6 months (14.5%), and more than six months (79.5%) after transplantation. CMV monitoring is made once a week until the patient is discharged from the hospital. Then the patients were monitored every 15 days for the presence of CMV-pp65 antigenemia between one and four months following transplantation—the period of higher risk for CMV infection—and then every month in within 4 to 12 months post-transplantation.

Data gathered included age of recipient and donor, gender of recipient and donor, type of donor, time of CMV infection after transplantation, trough and 2-hour post-dose levels of cyclosporine, renal allograft function, hemoglobin concentration, fasting blood sugar, liver enzymes and lipid profile.

CMV Antigen Assay 

All blood samples taken from patients were assayed for CMV-pp-65 antigenemia. CMV infection was defined as the detection of CMV-pp65 antigen in peripheral blood leukocytes (mostly polymorphonuclear cells) using a cocktail of two monoclonal antibodies (C10/C11) directed against pp65. The CMV antigenemia assay was performed with the CMV Brite Turbo kit (IQ Products, Groningen, the Netherlands), according to the manufacturer’s instructions. Briefly, 3–5 mL of venous blood sample collected in EDTA-treated tubes were processed within six hour after the sample was drawn. The leukocytes were isolated from peripheral blood, counted and spotted onto a glass slide. Following incubation with monoclonal antibodies (C10/C11) directed against CMV-pp65, cells were stained with substrate and visualized for typical nuclear staining. CMV-pp65-positive cells were counted using an immunofluorescence microscope at 1000´ magnification. A positive assay was defined by the presence of at least one positively stained leukocyte on the slide; positively stained cells were expressed as the number of fluorescing cells per 150,000 leukocytes examined. 

Statistical Analysis 

Statistical analyses were done by SPSS^®^ for Windows^®^ ver 17.0. Continuous variables are expressed as mean±SD. One-sample Kolmogorov-Smirnov test showed that all variables in this study did not have normal distribution, thus, non-parametric methods were used for analyses of data. Comparisons of qualitative variables were performed by χ^2^ test. Multivariate logistic regression analysis was used to determine the independent risk factors associated with CMV infection. Variables that were significant at the p<0.2 univariate level were included in the multivariate analysis. All tests were two tailed, and p values <0.05 were considered statistically significant. 

Ethics 

This study was approved by the local Ethics Committee of Baqiyatallah University.

## Results

Sixty-three percent of studied patients were males. The mean±SD age of participants was 38±15 (range: 6–84) years. The majority of donors were male (81.2%). The majority of recipients (81%) received a kidney from a living unrelated donor (LURD), 9% from living related donor (LRD), and 10% of patients from deceased donors. All patients received triple immunosuppressive regimens consisting of cyclosporine, mycophenolate mofetile/azathioprine, and prednisolone.

The demographic data of the study participants are shown in Table 1. No significant differences were observed in terms of gender and age of recipients and donors between the two studied groups (Table 1).

**Table 1:** Demographic data and univariate analysis results

**Variables**	**CMV positive patients**	**CMV negative patients**	**p value**
**Age of recipient (yr)**	40±16	38±15	0.1
**Age of donor (yr)**	29±8	29±7	0.3
**Cyclosporine trough level (ng/mL)**	267±134	187±121	<0.001
**Two post-dose level of cyclosporine (ng/mL)**	777±219	598±217	<0.001
**Serum creatinine (mg/dL)**	1.56±0.85	1.58±1.13	0.016
**Fasting blood glucose (mg/dL)**	113±67	103±47	0.005
**Hemoglobin concentration (g/dL)**	11.6±2.0	12.0±2.3	0.02
**Alanine aminotransferase (IU/L)**	54±97	46±74	<0.001
**Triglyceride level (mg/dL)**	207±84	193±66	0.007
**LDL-cholesterol level (mg/dL)**	110±42	104±38	0.09
**HDL-cholesterol level (mg/dL)**	44±17	47±17	0.001
**Time after Tx** **≤1 month** **2–6 months** **>6 months**	24% 52% 13%	76% 48% 87%	<0.001
**Recipient sex (M/F)**	61/39	64/36	0.2
**Donor sex (M/F)**	84/16	81/19	0.4

During the studied period, 755 CMV-Ag-positive samples were detected in 671 (21.9%; 95% CI: 20.4%–23.4%) RTRs. The majority of infected patients (n=603) responded to treatment with no recurrence, while recurrence of CMV infection occurred in only 68 patients (one episode in 61, two in six recipients and three in another patient).

The CMV infection rate was higher within 2–6 months of kidney transplantation (52%) than during other two periods (Table 1). The rate of CMV-Ag positivity in the first month of transplantation was more than twice that observed six months of transplantation (Table 1). The rate of CMV infection was higher in patients received kidney from deceased donors—one-fourth of patients who received kidneys from deceased donors became CMV-Ag positive. The rate was significantly (p=0.006) higher than those observed in LRD (13%) and LURD (13.7%) transplant recipients.

The serum cyclosporine levels were significantly higher in CMV-Ag-positive patients than those with no infection (Table 1). Patients with CMV infection had higher fasting blood glucose than CMV-Ag-negative cases (Table 1). There was a significant correlation between renal allograft dysfunction and CMV infection (Table 1). The CMV-Ag-positive patients had also lower hemoglobin concentration, higher ALT level, higher TG concentration, lower HDL alevel and higher LDL level than patients with no infection (Table 1).

In multivariate analysis, time since kidney transplantation (p<0.001) and renal allograft dysfunction (p<0.001) were the only risk factors associated with CMV infection (Table 2).

**Table 2:** Risk factors associated with CMV infection in multivariate analysis

**Variable**	**OR (95% CI)**
Time after transplantation	0.156 (0.066–0.370)
Cyclosporine through level	0.993 (0.984–1.002)
Two post-dose level of cyclosporine	0.999 (0.994–1.003)
Serum creatinine level	0.215 (0.101–0.456)
Fasting blood glucose	0.996 (0.988–1.003)

## DISCUSSION

Our results showed that the period with the highest risk of CMV infection was from the second to the sixth months of kidney transplantation (52%). CMV infection occurred in the majority of our recipients within the first six post-transplantation months when immunosuppression was at maximal intensity. The incidence of CMV in the kidney transplant recipients is reported to be between 8% and 32% [8]; it usually occurs in the first six months after transplantation [9]. The overall incidence of CMV infection in the current study was 21.9% (95% CI: 20.4%–23.4%). Similarly, Pourmand, *et al*, reported a rate of 17.6% of CMV infection in 172 renal transplant patients followed at a single center in Iran [10].

In the present study, the incidence of infection was directly related to the immunosuppression dose used. The CMV-Ag-positive patients had a higher cyclosporine blood levels compared to those who had no infection. Similarly, Alimagham, *et al*, in a study on 511 RTRs reported that the rate of CMV infection was closely correlated to cyclosporine blood level [11]. In a small study, Monforte, *et al*, showed that higher cyclosporine blood levels in lung transplant recipients are associated with CMV infection [12]. In a series of 54 consecutive patients undergoing peripheral blood stem cell transplantation, cyclosporine levels were also higher in patients with CMV infection compared to non-infected individuals [13].

The rate of CMV infection in our study was higher in deceased kidney recipients, which is consistent with other reports [14,15]. For example, 62% of the living related kidney recipients developed CMV infection, while the infection was observed in 79% of the deceased kidney recipients [14].

It is of interest that recipients with CMV infection had higher level of fasting blood glucose than CMV-Ag-negative cases. Although, Wyzgal, *et al*, reported that no difference was observed in the incidence of CMV infection in both diabetic and non-diabetic patients (18.7% in diabetic patients *vs*. 21.7% in non-diabetic group) [16], they showed that early hyperglycemia accounted for higher rate of CMV infection [16].

The multivariate analysis of our data showed that CMV infection was also associated with worse renal allograft function. An association between CMV infection and renal allograft impairment has been previously reported [17,18]. 
